# A Novel IRAK4 Inhibitor DW18134 Ameliorates Peritonitis and Inflammatory Bowel Disease

**DOI:** 10.3390/molecules29081803

**Published:** 2024-04-16

**Authors:** Yuqing Huang, Yi Ning, Zhiwei Chen, Peiran Song, Haotian Tang, Wenhao Shi, Zhipeng Wan, Gege Huang, Qiupei Liu, Yun Chen, Yu Zhou, Yuantong Li, Zhengsheng Zhan, Jian Ding, Wenhu Duan, Hua Xie

**Affiliations:** 1 Zhongshan Institute for Drug Discovery, Shanghai Institute of Materia Medica, Chinese Academy of Sciences, Zhongshan 528400, China; huangyuqing202103@163.com (Y.H.); songpeiran@163.com (P.S.); tanghaotian0381@zidd.ac.cn (H.T.); shiwenhao54@163.com (W.S.); wswzp19911118@163.com (Z.W.); ge949856481@163.com (G.H.); zhouyu1823@163.com (Y.Z.); liyuantong547@zidd.ac.cn (Y.L.); 2 College of Pharmacy, Guizhou Medical University, Guiyang 561113, China; 3 Division of Antitumor Pharmacology & State Key Laboratory of Drug Research, Shanghai Institute of Materia Medica, Chinese Academy of Sciences, Shanghai 201203, China; ningyi_1995@163.com (Y.N.); qiupei.liu@nottingham.edu.cn (Q.L.); jding@simm.ac.cn (J.D.); 4 University of Chinese Academy of Sciences, Beijing 100049, China; s20-chenzhiwei@simm.ac.cn; 5 Small-Molecule Drug Research Center, Shanghai Institute of Materia Medica, Chinese Academy of Sciences, Shanghai 201203, China; 15251756609@163.com (Y.C.); zszhan@simm.ac.cn (Z.Z.); 6 Department of Chemical and Environment Engineering, Science and Engineering Building, The University of Nottingham Ningbo China, Ningbo 315100, China

**Keywords:** IRAK4 inhibitor, peritonitis, inflammatory bowel disease (IBD), macrophage

## Abstract

IRAK4 is a critical mediator in NF-κB-regulated inflammatory signaling and has emerged as a promising therapeutic target for the treatment of autoimmune diseases; however, none of its inhibitors have received FDA approval. In this study, we identified a novel small-molecule IRAK4 kinase inhibitor, DW18134, with an IC_50_ value of 11.2 nM. DW18134 dose-dependently inhibited the phosphorylation of IRAK4 and IKK in primary peritoneal macrophages and RAW264.7 cells, inhibiting the secretion of TNF-α and IL-6 in both cell lines. The in vivo study demonstrated the efficacy of DW18134, significantly attenuating behavioral scores in an LPS-induced peritonitis model. Mechanistically, DW18134 reduced serum TNF-α and IL-6 levels and attenuated inflammatory tissue injury. By directly blocking IRAK4 activation, DW18134 diminished liver macrophage infiltration and the expression of related inflammatory cytokines in peritonitis mice. Additionally, in the DSS-induced colitis model, DW18134 significantly reduced the disease activity index (DAI) and normalized food and water intake and body weight. Furthermore, DW18134 restored intestinal damage and reduced inflammatory cytokine expression in mice by blocking the IRAK4 signaling pathway. Notably, DW18134 protected DSS-threatened intestinal barrier function by upregulating tight junction gene expression. In conclusion, our findings reported a novel IRAK4 inhibitor, DW18134, as a promising candidate for treating inflammatory diseases, including peritonitis and IBD.

## 1. Introduction

Inflammation is a biological reaction involving blood vessels, immune cells, and inflammatory mediators [1], serving as the fundamental cause for a broad spectrum of diseases, including inflammatory bowel disease (IBD), chronic obstructive pulmonary disease (COPD), peritonitis, and rheumatoid arthritis (RA) [2]. The global increase in the incidence and mortality rates of inflammatory diseases has gradually positioned it as a significant threat to human life and health [3,4]. Traditional medications such as antibiotics or immunosuppressants have proven inadequate to meet the clinical demands. Consequently, novel therapeutic approaches for alleviating inflammation focus on regulating the kinase activity produced by inflammatory mediators [5]. Despite extensive clinical research on kinase inhibitors targeting the immune pathways, including BTK, JAK, and IRAK4, breakthroughs in an effective therapeutic drug remain elusive.

Peritonitis is a prevalent inflammatory disease characterized by clinical manifestations such as diarrhea, abdominal pain, abdominal infection, and multiple organ failure, with a mortality rate exceeding 60% [6,7]. Despite the incomplete elucidation of the pathogenic mechanisms underlying this disease, extensive reported research suggests a close association between peritonitis and macrophages [8,9]. Throughout the development of peritonitis, macrophages produce cytokines such as tumor necrosis factor (TNF)-α and interleukin-6 (IL-6), mediating and promoting pro-inflammatory responses [10]. Currently, the primary drugs for clinically treating peritonitis include glucocorticoids and immunosuppressants. However, due to the low specificity of these drugs, they are prone to causing widespread systemic adverse reactions [11]. Therefore, there is an urgent need to develop novel drugs targeting peritonitis.

Inflammatory bowel disease (IBD) is another common inflammatory disease with non-infectious, chronic, and recurrent characteristics. Its primary manifestation involves recurrent episodes of prolonged diarrhea and abdominal pain, encompassing ulcerative colitis (UC) and Crohn’s disease (CD) [12]. In the early twentieth century, the disease was primarily prevalent in European regions, accounting for 0.5% of the total population in the Western world. However, the prevalence and incidence of IBD have been increasing over the years, transforming it into a global health issue [13,14]. IBD not only impacts patients’ quality of life but also increases their risk of developing colon cancer. The etiology of IBD is not fully understood, but it is generally believed to be associated with various factors such as genetic, environmental, microbiological, and inflammatory causes. Among these factors, disorders of the immune system play a predominant role in the IBD pathogenesis [15,16]. Research indicates the presence of a bounty of macrophages in the mucous membranes of patients with IBD, with the secretion of multiple cytokines, including IL-6 and TNF-α [17], confirming the crucial role of immune system disorders in the onset of IBD. The occurrence of IBD is typically accompanied by specific clinical symptoms, such as weight loss, diarrhea, colon shortening, and edema [18]. Conventional treatments such as salicylic acid, steroid hormones, and immunosuppressive agents have limited efficacy and severe side effects [19]. Therefore, there is an urgent need for in-depth research into more effective molecular mechanisms and the identification of suitable alternative drugs to combat IBD.

Interleukin receptor-associated kinase 4 (IRAK4) is a serine/threonine kinase consisting of an N-terminal death domain, a hinge region, and a C-terminal kinase structural domain. IRAK4 is an important mediator in the signal transduction of Toll-like receptors (TLRs) and interleukin-1 receptor (IL-1R) by binding the adaptor protein myeloid differentiation factor 88 (MyD88) and inducing signaling through IRAK1 and IRAK2 [20] to form myddosome. Myddosome assembly promotes the downstream activation of NF-κB, mediating the expression of inflammatory factors and fostering inflammatory progression [21]. The deletion or inactivation of IRAK4 activity in mice has been shown to prevent inflammation in multiple inflammatory disease models [22]. Notably, a couple of IRAK4 inhibitors have been employed in the clinical treatment of inflammation and autoimmune diseases. Specifically, positive clinical data have been reported with IRAK4 inhibitor PF-06650833 for the first time in patients with RA [23]. Thus, the development of novel inhibitors targeting IRAK4 is a promising approach for treating various immune-related diseases.

In this study, we developed a novel IRAK4 inhibitor, DW18134, which exhibited considerable anti-inflammatory activity in vitro and alleviated symptoms of acute peritonitis and IBD in vivo. These observed effects were primarily attributed to the ability of DW18134 to inhibit the IRAK4-induced activation of NF-κB signaling, subsequent macrophage infiltration, and inflammatory cytokine release.

## 2. Results

### 2.1. DW18134 Potently Inhibited IRAK4 Kinase Activity and Attenuated the LPS-Induced IRAK4 Signaling Transduction and Secretion of Cytokines in Macrophages

**Figure 1:**
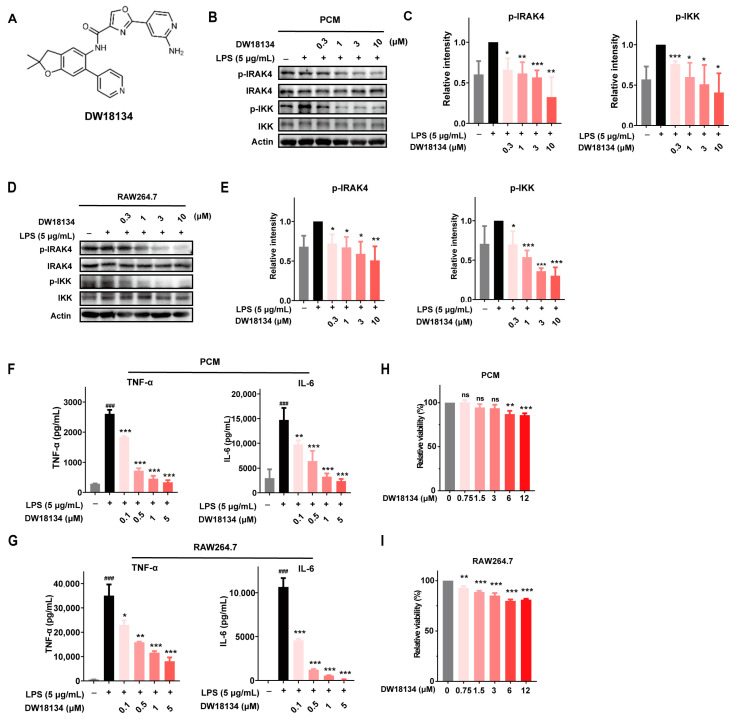
Chemistry structure of compound DW18134 and its effects on the LPS-induced IRAK4 signaling transduction and secretion of cytokines in macrophages. (**A**) Chemical structure of DW18134. (**B**) Representative immunoblots of p-IRAK4 and p-IKK in PCM cells that were stimulated with LPS (5 µg/mL) and treated with or without indicated concentrations of DW18134 for 2 h. (**C**) Quantification of p-IRAK4 and p-IKK levels in PCM cells. (**D**) Representative immunoblots of p-IRAK4 and p-IKK in RAW264.7 cells that were stimulated with LPS and treated with or without DW18134. (**E**) p-IRAK4 and p-IKK levels in RAW264.7 cells were quantified. (**F**,**G**) Secretion levels of TNF-a and IL-6 in the supernatants of PCM and RAW264.7 cells were measured by ELISA. (**H**,**I**) Following the treatment of DW18134 for 24 h, cell viability of PCM and RAW 264.7 cells was determined by CCK-8 assays. A representative of three independent repeats is shown in (**B**,**D**). Data represent the mean ± standard deviation (SD) from three independent biological replicates in (**C**,**E**–**I**). * *p* < 0.05, ** *p* < 0.01, *** *p* < 0.001 vs. LPS; ### *p* < 0.001 vs. control. ns: no significance.

IRAK4 kinase has been reported to be closely associated with inflammatory and autoimmune diseases, and several IRAK4 inhibitors have been used in the study of such disorders [23,24,25]. Therefore, we aimed to develop a novel IRAK4 inhibitor to combat inflammatory diseases. We have developed a series of IRAK4 inhibitors bearing an oxazole-4-carboxamide scaffold via structure-based drug design (SBDD) [26], among which DW18134 outperformed its counterparts as a potent and selective IRAK4 inhibitor (Figure 1A). The enzymatic activity assay demonstrated that DW18134 effectively inhibited IRAK4, with an IC_50_ value of 11.2 nM, which was less potent than that of positive control compound PF-06650833 (Table 1).

As IRAK4 regulates the TLR/Ll-1R receptors on macrophages, transmits downstream signals, and participates in the regulation of innate immunity and inflammation [27], the in vitro anti-inflammatory activity of DW18134 was evaluated using mouse primary peritoneal macrophages (PCM) and the RAW264.7 macrophage cell line. As shown in Figure 1B,C, DW18134 dose-dependently inhibited LPS-induced IRAK4 activation and downstream IKK phosphorylation; the inhibition rates at a 10 μM concentration were 46.5% and 65.3% in PCM cells, respectively. Consistent results were obtained when the same experiment was performed on RAW264.7 cells. DW18134 significantly downregulated the phosphorylation of IRAK4 and its downstream molecule IKK in RAW264.7 cells (Figure 1D,E).

Since the TLR/MYD88 pathway involved in IRAK4 affects inflammatory cytokines, such as TNF-α and IL-6 secretion, we examined the levels of factor secretion on the supernatants of macrophages using an enzyme-linked immunosorbent assay (ELISA). The results showed that DW18134 inhibited TNF-α and IL-6 secretion in a dose-dependent manner in PCM (Figure 1F) and RAW264.7 cells (Figure 1G). Meanwhile, we examined the cell viability of DW18134 on macrophages, and the results showed that DW18134 did not affect the proliferation of macrophages, which excluded the possible effect of the drug on cell killing that produced the above results (Figure 1H,I).

### 2.2. DW18134 Significantly Decreased LPS-Induced Inflammatory and Pathological Injury in the Mice Peritonitis Model

**Figure 2:**
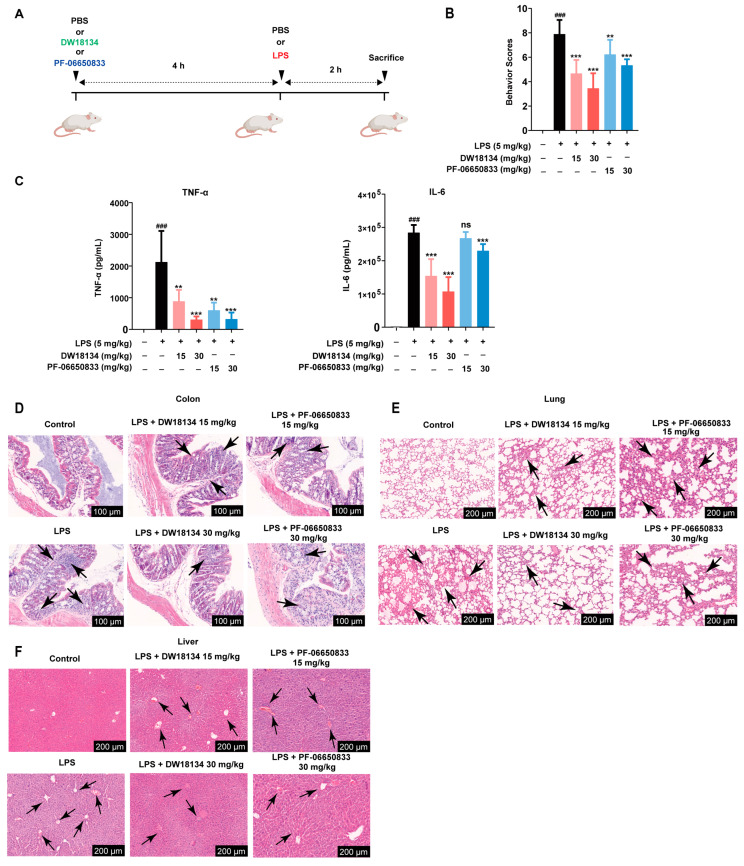
DW18134 ameliorated inflammatory phenotype and acute tissue damage in LPS-induced peritonitis mice (*n* = 8). (**A**) Schematic illustration of the experimental strategy. Balb/c mice were pretreated with DW18134 or PF-06650833 or phosphate buffer saline (PBS) for 4 h and then challenged with PBS or LPS (5 mg/kg) through i.p. administration for 2 h. Following the induction, mice were sacrificed, and serum samples were collected. (**B**) Scores on behavior were observed. (**C**) The levels of TNF-a and IL-6 in the serum of mice with peritonitis were measured using ELISA. (**D**–**F**) Representative H&E-stained sections indicated the pathological damage in the colons, lungs, and livers of mice with peritonitis. The arrows in Figure (**D**) indicate inflammatory cell infiltration. The arrows in Figure (**E**) indicate infiltrations around blood vessels and the alveolar cavity. The arrows in Figure (**F**) indicate hepatic filament disorganization and fibrosis in the liver. ** *p* < 0.01, *** *p* < 0.001 vs. LPS; ### *p* < 0.001 vs. control. ns: no significance.

As DW18134 exhibited significant anti-inflammatory effects in vitro, the in vivo activity was evaluated, using PF-06650833 as a control. We first evaluated the efficacy of DW18134 in LPS-induced peritonitis in mice. Figure 2A depicts a schematic representation of the experimental protocol. Balb/c mice were primarily treated with DW18134 or PF-06650833 before LPS administration (Figure 2A). The general behavior of mice was observed during the experiment, and the scoring criteria are detailed in Table S1 [28]. The results demonstrated a significant increase in the behavior scores of mice with LPS compared to those in the control group, while the increased score induced by LPS was dramatically and dose-dependently reversed upon DW18134 treatment at doses of 15 mg/kg and 30 mg/kg (Figure 2B). The serum of mice was further analyzed for levels of inflammatory factors. High levels of TNF-α and IL-6 were detected in the serum of LPS-treated mice, and DW18134 treatment with LPS significantly suppressed the secretion of TNF-α and IL-6 in a dose-dependent manner. Intriguingly, the effects of DW18134 on the LPS-induced behavior score and cytokine level in mice serum surpassed that of the positive control drug PF-06650833 (Figure 2B,C).

LPS-induced acute peritonitis typically results in diverse levels of intestinal and hepatic impairment in mice, with acute lung injury being a prevalent concomitant condition [29,30]. Therefore, the mice were euthanized to extract their colons, lungs, and livers for pathological examination. The LPS group exhibited significant inflammatory cell infiltration in the colon area (Figure 2D) and notable damage to lung structures, such as interstitial expansion, fibrosis, and the aggregation of inflammatory mononuclear cells in comparison to the control group (Figure 2E). Treatment with increasing doses of DW18134 resulted in a considerable improvement in both colon and lung structure damage, and the effect was better than that of PF-06650833 (Figure 2D,E). Notably, in the high-dose group (30 mg/kg), the lung structure was similar to that of the control group, indicating potential protective abilities of DW18134 against pulmonary injury (Figure 2E). The liver histopathological findings in the LPS group revealed disorganized hepatic filaments, fibrous hyperplasia in the portal region, and a limited presence of inflammatory cell infiltration. DW18134 exhibited significant amelioration of LPS-induced liver injury, and the drug effect was significantly better than the positive control (Figure 2F).

These results collectively indicated that DW18134 possessed the potential to enhance the activity status of mice with peritonitis, reduced the secretion of inflammatory factors, and alleviated acute damage to the intestinal tract, lungs, and liver.

### 2.3. DW18134 Reduced Macrophage Infiltration and Pro-Inflammatory Gene Expression and Regulated IRAK4 Signaling Pathway Activation in LPS-Induced Peritonitis Mice

**Figure 3:**
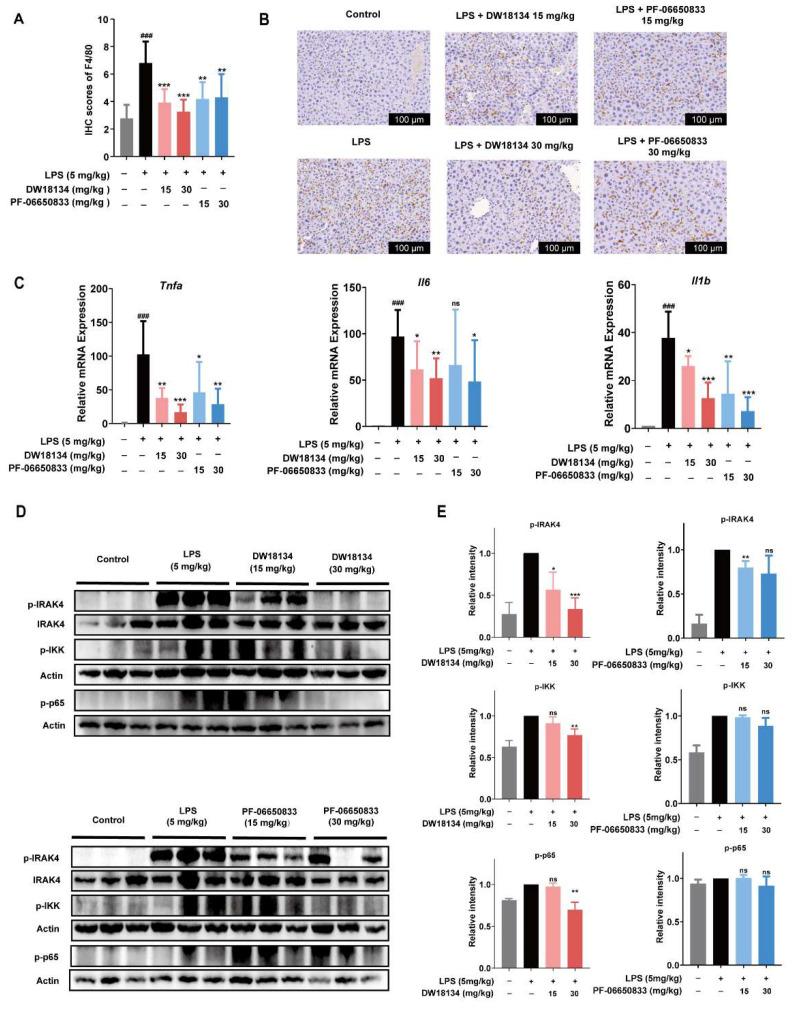
DW18134 reduced macrophage infiltration and pro-inflammatory gene expression and abrogated IRAK4 signaling pathway activation in LPS-induced peritonitis mice (*n* = 8). Immunohistochemical staining of macrophage infiltration in the liver of mice with peritonitis. Includes quantitative data (**A**) and representative images (**B**). (**C**) *Tnfa*, *Ill6*, and *Il1b* gene expression from liver homogenates measured by RT-qPCR. (**D**) Expression of p-IRAK4, p-IKK, and p-p65 in liver tissue of peritonitis mice treated with DW18134 or PF-06650833. (**E**) Quantification of p-IRAK4, p-IKK, and p-p65 levels in liver tissue based on three independent biological replicates. Data are expressed as mean ± SD. * *p* < 0.05, ** *p* < 0.01, *** *p* < 0.001 vs. LPS; ### *p* < 0.001 vs. control. ns: no significance.

Based on pathological findings (Figure 2F), we speculated that peritonitis mice have hepatic macrophage infiltration and increased expression of inflammatory factors. To confirm this assumption, we examined macrophage infiltration by immunohistochemistry. As shown in Figure 3B, the brown portion represents the macrophage marker F4/80, while Figure 3A shows the intensity score of the region in Figure 3B. F4/80 was expressed in more significant number and intensity in the model group. However, DW18134 treatment significantly reduced the number and intensity of macrophage infiltration, especially with a 50% reduction in infiltration under treatment at 30 mg/kg (Figure 3A,B).

Acute liver injury induced by LPS is typically characterized by an increase in the expression of inflammatory factor genes [29]. DW18134 significantly reduced the LPS-stimulated expression of *Tnfa*, *Il6,* and *Il1b* in peritonitis mice livers; the effect showed good dose dependence at 15 mg/kg and 30 mg/kg (Figure 3C). Additionally, we confirmed the targeting effect of DW18134 on IRAK4 in vivo. The results indicated that DW18134 effectively targeted IRAK4 and downregulated its phosphorylation level. Furthermore, it significantly inhibited the downstream activation of IKK and p65 (Figure 3D,E). The expression levels of p-IRAK4 and p-p65 were almost identical to those of the blank group at the administered dose of 30 mg/kg (Figure 3D,E). In conclusion, DW18134 could potentially treat acute peritonitis by reducing macrophage infiltration, regulating the NF-κB signaling pathway through targeting IRAK4, and subsequently inhibiting the expression of inflammatory cytokines to ameliorate pathological injury.

### 2.4. DW18134 Effectively Ameliorated Symptoms and Colonic Inflammatory Injury of DSS-Induced Colitis in Mice

**Figure 4:**
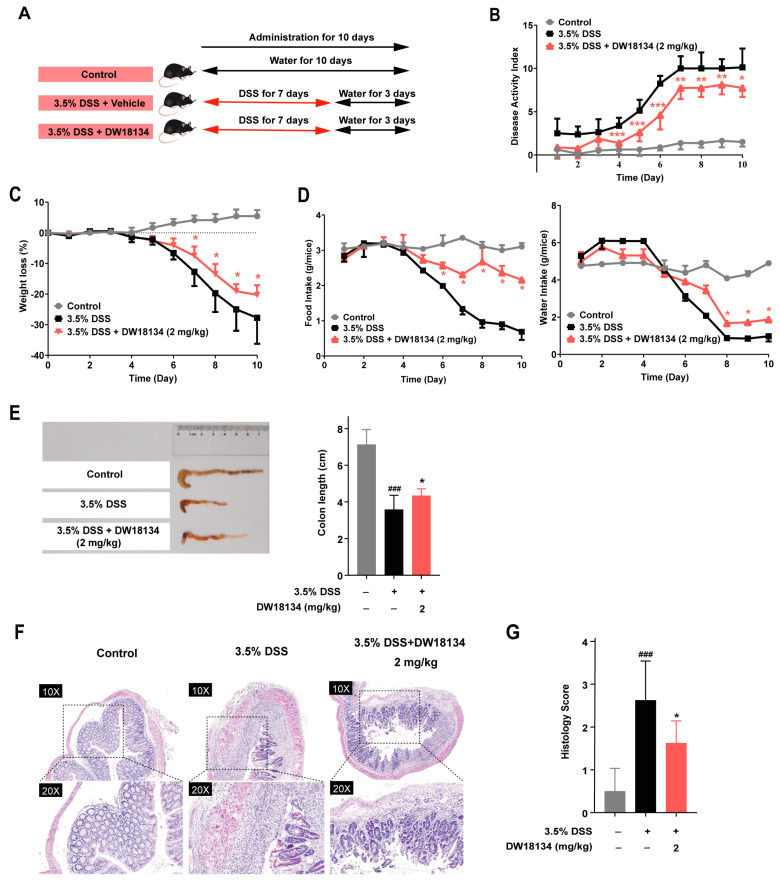
DW18134 ameliorated symptoms and reduced intestinal pathological damage in mice with DSS-induced experimental colitis (*n* = 6). (**A**) Schematic illustration of the experimental regime. Mice were induced by oral administration of DSS from day 0 to day 7, with the normal control group receiving only normal drinking water. DW18134 (2 mg/kg) was i.p. from day 1 until the end of the experiment. (**B**,**C**) Disease activity index (DAI) and body weight loss (% initial body weight) were monitored at the indicated time points. (**D**) Daily observations were made on food and water intake. (**E**) Representative images of colon morphology and quantitative colon length. (**F**,**G**) Representative images of H&E staining of colon sections and histological scores. * *p* < 0.05, ** *p* < 0.01, *** *p* < 0.001 vs. 3.5%DSS; ### *p* < 0.001 vs. control. ns: no significance.

We further investigated the in vivo anti-inflammatory activities of DW10134 in dextran sulfate sodium (DSS)-induced colitis mouse models [31]. The mice were subjected to oral administration of drinking water containing 3.5% DSS for 7 consecutive days, followed by drinking water for 3 days. They received intraperitoneal injections of DW18134 at a dosage of 2 mg/kg daily from day one until the experimental endpoint (Figure 4A).

As illustrated in Figure 4B,C, a remarkable increase in the disease activity index (DAI) and body weight loss in DSS-treated mice was observed starting from day 5 compared to the control group. In contrast, DW18134 (2 mg/kg) potently ameliorated the experimental symptoms of IBD, manifested with reversed body weight loss, diarrhea, and stool bleeding (Figure 4B,C). Furthermore, DW18134 effectively ameliorated the symptoms associated with reduced food intake and water consumption in IBD mice (Figure 4D). Moreover, colon lengths of DW18134-treated mice were significantly longer than those of DSS-treated mice (Figure 4E). Histologically, DSS induced severe epithelial damage, goblet cell and crypt loss, dense inflammatory cell infiltration, and mucosal ulcers [32], whereas DW18134 largely prevented colonic tissue injury (Figure 4F,G). These findings suggested that DW18134 effectively alleviated symptoms, including weight loss, diarrhea, and blood in stool, and enabled the revelation of an increase in food and water intake in DSS-induced IBD mice.

### 2.5. DW18134 Reduced Colonic Macrophage Infiltration and Inflammatory Factor Expression and Restored Intestinal Barrier in Mice with DSS-Induced Colitis

**Figure 5:**
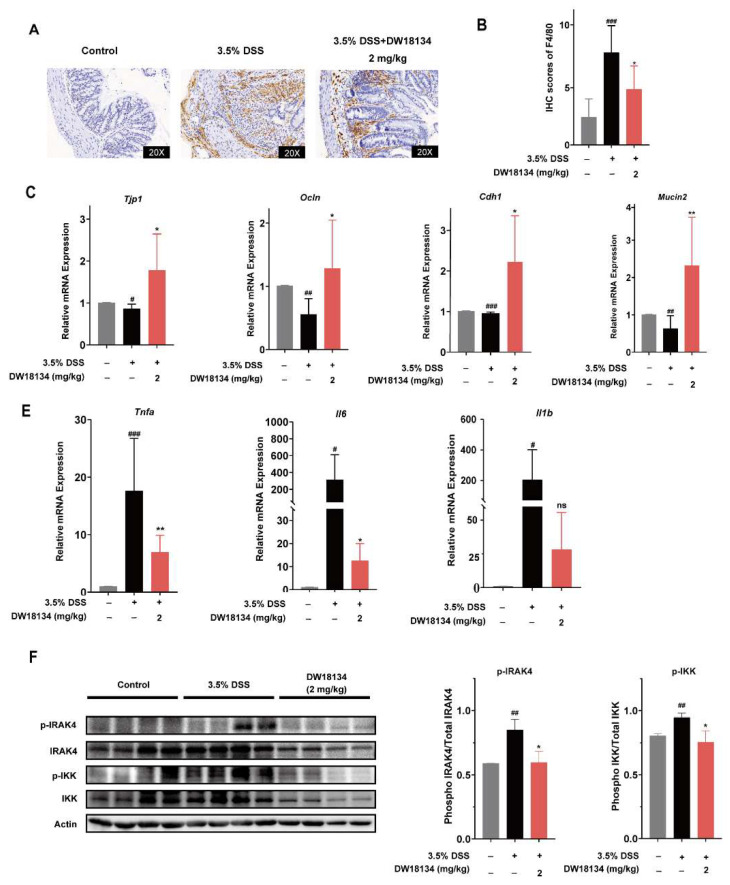
DW18134 reduced macrophage infiltration and the expression of inflammatory factors and restored the intestinal barrier in mice with DSS (*n* = 6). (**A**,**B**) Immunohistochemistry was used to detect macrophage infiltration in the colorectum. (**C**,**D**) Gene expression levels of tight junction proteins and Mucin2 were detected in the colon of IBD mice by RT-qPCR. (**E**) The mRNA expression of cytokines *Tnfa*, *Il6*, and *Il1b* in colonic tissues. (**F**) Representative immunoblots of p-IRAK4 and p-IKK in the colonic homogenates from each group. p-IRAK4 and p-IKK levels were quantified (right panel). Quantified data represent the mean ± SD from three independent biological replicates. * *p* < 0.05, ** *p* < 0.01 vs. 3.5%DSS; # *p* < 0.05, ## *p* < 0.01, ### *p* < 0.001 vs. control. ns: no significance.

In DSS-induced colitis, an increase in macrophage infiltration and pro-inflammatory mediators [33], as well as barrier dysfunction, can be observed [34]. We first investigated macrophage infiltration in intestinal tissues at the pathological tissue level. The level of macrophage F4/80 positively expressed infiltration was significantly increased in the colon tissue of the DSS-treated group compared to the control group, which can be significantly decreased in the DW18134-treated group, suggesting that DW18134 reduced macrophage infiltration in DSS-induced mice (Figure 5A,B).

Tight junctions regulate paracellular permeability and maintain intestinal barrier function through transmembrane proteins (e.g., Occludin and claudins) and peripheral membrane proteins (e.g., ZO-1), and the disruption of tight junction proteins can directly lead to intestinal barrier dysfunction [35]. Our RT-qPCR results demonstrated a significant downregulation of colonic tight junction proteins (including ZO-1, E-cadherin, and Occludin) in DSS mice compared to normal mice. Importantly, this observed phenomenon was effectively reversed by the administration of DW18134 (Figure 5C). Furthermore, mucin 2, also known as MUC2, is secreted from goblet cells, contributing to the chemical barrier in the gut [36]. In keeping with the loss of goblet cells (Figure 4F), reduced MUC2 in colonic homogenates was monitored in vehicle mice and obviously reversed upon DW18134 treatment (Figure 5D). These findings indicated that DW18134 has the potential to effectively mitigate IBD by attenuating macrophage infiltration and restoring the expression of tight junction proteins.

The increased production of pro-inflammatory mediators, such as IL-1β, IL-6, and TNF-a, plays a vital role in the development of DSS-induced IBD. We found that DW18134 significantly suppresses the gene expression of *Tnfa, Il6*, and *Il1b* in colon tissues, reducing *Tnfa* levels by half and *Il6* and *Il1b* levels by nearly 10-fold compared to the DSS group (Figure 5E). These findings indicated that DW18134 effectively reduces inflammatory gene expression. Furthermore, we confirmed the targeting efficacy of DW18134 in IBD mice and observed its significant inhibition on IRAK4 along with IKK activation induced by DSS stimulation. In conclusion, DW18134 exhibited potential for attenuating macrophage infiltration, restoring intestinal barrier integrity, modulating the NF-κB pathway through the IRAK4 targeting mechanism, and suppressing inflammatory factor secretion to combat IBD.

## 3. Materials and Methods

### 3.1. Compounds

Compound DW18134 was designed and synthesized by Dr. Wenhu Duan’s laboratory [26]. PF-06650833 was purchased from MedChemExpress (#HY-19836, Princeton, NJ, USA). All compounds were dissolved in dimethyl sulfoxide (DMSO, Sigma-Aldrich, Burlington, MA, USA) at the concentration of 10 mM and stored at −20 °C. For in vivo experiments, DW18134 and PF-06650833 were dissolved in saline (SINOPHARM, Beijing, China).

### 3.2. Reagents and Antibodies

All tissue culture materials were purchased from Gibco™, New York, NY, USA, ThermoFisher Scientific, Waltham, MA, USA, CST, Boston, MA, USA, and PEproTech, Cranbury, NJ, USA. Different gene-specific primers were procured from Suzhou GENEWIZ Biotechnology Co., Ltd., Suzhou, China. The detailed sequences of the gene-specific primers are shown in Table S2.

### 3.3. Cell Culture and Treatments

Mouse RAW264.7 macrophage cells were procured from ATCC. Primary mouse peritoneal macrophages were cultured in Roswell Park Memorial Institute 1640 (RPMI1640) medium containing penicillin (100 U/mL) and streptomycin (100 μg/mL) and supplemented with 10% fetal bovine serum (FBS) in a humidified 5% CO_2_ environment at 37 °C. RAW264.7 macrophage cells were cultured in Dulbecco’s Modified Eagle Medium (DMEM) containing penicillin (100 U/mL) and streptomycin (100 μg/mL) and supplemented with 10% FBS in a humidified 5% CO_2_ environment at 37 °C. Cells were pretreated with DW18134 for 2 h followed by the incubation of LPS (1 μg/mL) for 2 or 24 h. Upon the termination of incubations, cells were washed twice with ice-cold PBS and harvested with a trypsin–EDTA solution using a cell scraper.

### 3.4. Cell Viability Assay

RAW264.7 cells and mouse primary peritoneal macrophages were seeded in 96-well culture plates at an appropriate density for 12 h and were then supplemented with different concentrations of DW18134. After incubation for 24 h, cell viability was measured by a CCK-8 assay according to the manufacturer’s instructions.

### 3.5. Western Blot Analysis and Antibodies

Cells were harvested and lysed with SDS lysis buffer and heated at 100 °C for 15 min. Whole-cell lysate samples were resolved on SDS-PAGE gels and transferred to nitrocellulose membranes. To probe for different proteins of varying molecular weights in the blots, the membranes were cut horizontally. After blocking the membranes in 5% nonfat milk for 1 h, the membranes were incubated with the respective antibodies for 12 h at 4 °C. The antibody for Phospho-IRAK4 (Thr345/Ser346) (D6D7) Rabbit mAb(dilution, 1:1000; #11927), IRAK4 antibody (dilution, 1:1000; #4363), Phospho-IKKα/β (Ser176/180) (16A6) Rabbit mAb(dilution, 1:1000; #2697), NF-κB p65 (D14E12) Rabbit mAb(dilution, 1:1000; #8242), Phospho-NF-κB p65 (Ser536) (93H1) Rabbit mAb(dilution, 1:1000; #3033), and actin (dilution, 1:20,000; # 60008-1-Ig, Proteintech, Cranbury, NJ, USA) were purchased from Cell Signaling Technology (CST, Boston, MA, USA). Secondary antibodies (1:2000; # 111-035-003, Jackson) for 1 h at room temperature.

Tissues were lysed with RIPA lysis buffer (#P0013B, Beyotime, Shanghai, China) supplemented with the protease inhibitor cocktail (#4693132001, Roche, Basel, Switzerland) and phosphatase inhibitor (#04906837001, Roche). For sample normalization, protein concentrations were determined using the bicinchoninic acid (BCA)-reagent-based protein quantitation assay kit (#23227, ThermoFisher Scientific). Equal amounts of protein were loaded on SDS-PAGE gels for blotting.

### 3.6. Real-Time Quantitative PCR

Total RNA was isolated from colon and liver tissues using a FastPure Cell/Tissue Total RNA Isolation Kit V2, and cDNA was synthesized using a cDNA HiScript II Q RT SuperMix for qPCR (+gDNA wiper) kit. To quantify mRNA expression, real-time PCR amplification was conducted using the CFX Connect^TM^ real-time PCR detection system (Bio-Rad, Hercules, CA, USA). The PCR products were detected with a ChamQ Universal SYBR qPCR Master Mix kit. The forward and reverse primer pairs are listed in Supplementary Table S2. The expression of mRNA was normalized using mouse actin mRNA.

### 3.7. Enzyme-Linked Immunosorbent Assay (ELISA)

After the cells were stimulated with DW18134 or LPS, culture supernatants were collected and used for ELISA assays. Also, we measured TNF-α and IL-6 cytokine levels in the serum samples of control and treated mice using mouse TNF-α and mouse IL-6 ELISA kits (ThermoFisher Scientific) following the manufacturer’s instructions.

### 3.8. Animals and Treatments

Male BALB/c mice (6–8 weeks, 19–22 g) and wild-type, male C57BL/6 (6–8 weeks, 19–22 g) mice were obtained from BesTest Biotechnology, Guangdong, Ltd. All mice were housed under specific pathogen-free conditions. All experiments were performed according to the guidelines of the Association for Assessment and Accreditation of Laboratory Animals Care International, where the animals were housed for 7 days in a 12 h light/dark cycle at 23 ± 2 °C with a relative humidity of 55 ± 5% and fed a regular rodent pellet diet and water ad libitum. All of the procedures were carried out strictly in accordance with the animal care and use protocol (202310001) approved by the Institutional Animal Care and Use Committee (IACUC) at the Zhongshan Institute of Drug Discovery.

### 3.9. Induction of DSS-Induced Experimental Colitis in Mice and Drug Treatment

IBD was induced via administration of 3.5% (*w*/*v*) DSS in drinking water. Mice received either regular drinking water (normal control) or DSS drinking water (model) for 7 days, followed by regular drinking water for 3 days as described previously [32]. Mice were randomly divided into 3 groups: untreated normal control, DSS control, and DW18134 (2 mg/kg) treated. DW18134 mice were intraperitoneally injected once daily for 10 d (Figure 4A).

### 3.10. Disease Activity Index (DAI)

To evaluate the disease activity index (DAI), body weight, fecal consistency, and gross blood in feces were observed daily: DAI scores = weight loss rate score + fecal consistency score + blood in stool score [37].

### 3.11. Induction of LPS-Induced Peritonitis in Mice and Drug Treatment

In the present study, vehicle control or DW18134 (15 mg/kg/30 mg/kg) was administered orally. After 4 h, mice were challenged with i.p. injection of saline or LPS (5 mg/kg) for 2 h [38]. Mice were euthanized, and serum, liver, colon, and lung samples were collected. Serum samples were used to analyze TNF-α and IL-6 pro-inflammatory cytokines levels by ELISA. Liver, colon, and lung samples were isolated from these mice and used for H&E analysis, and the liver was used for RT-qPCR analysis and immunoblotting.

### 3.12. Hematoxylin and Eosin

Peritonitis mice were euthanized after 2 h of LPS stimulation, and parts of the livers, colons, and lungs were fixed in a 4% paraformaldehyde solution and embedded in paraffin to provide sections for histological evaluation. The experimental operation was performed according to the kit protocol.

IBD mice were sacrificed at the end of the experiment. The entire colon was dissected and flushed with ice-cold PBS. The severity of colitis was evaluated in sections stained with H&E blinded to the experimental conditions according to the criteria published by Santucci et al. [39], detailed in Table S3.

### 3.13. Immunohistochemistry Analysis

Paraffin-embedded tissue sections underwent antigen retrieval, endogenous peroxidase blocking, and primary antibody incubation overnight at 4 °C. Slides were rinsed again and then treated with goat anti-rabbit for 30 min at 37 °C. Sections were stained with DAB and counterstained with hematoxylin. Images were captured on a Slide Scan System, Shenzhen Shengqiang Technology Co., Ltd., Shenzhen, China, (SQS-600Pro). Quantitative analysis was performed with ImageViwer. Primary antibodies against F4/80 (#70076S) were purchased from Cell Signaling Technology (CST, Boston, USA), and the peroxidase-based immunohistochemistry detection kit (#AR1108, Boster, Wuhan, China), 3,3′-diaminobenzidine (DAB) (#DA1010, Solarbio, Beijing, China), antigen retrieval buffer (pH 9.0) (#YJ0023, Yongjin, Guangzhou, Biotechnology Co., Ltd., Guangzhou, China), and antibody diluent (#ZLI-9028, CST) were obtained from Sigma.

### 3.14. Statistical Analysis

All data were derived from at least three independent experiments, and statistical analyses were conducted using Graphpad 9.5.0 software. A densitometric analysis of Western blot data was performed using ImageJ 1.46r software. Data were analyzed by an unpaired Student’s T test, where the *p*-value indicated significance. All values were means ± SD. A level of *p* < 0.05 was considered significant.

## 4. Discussion

Peritonitis and IBD are prevalent inflammatory diseases characterized by excessive inflammation, resulting in acute organ injury and potential multi-organ failure, ultimately posing a threat to life [40,41]. In this process, the inflammatory mediators secreted by macrophages have been identified as crucial players in the pathological processes [5,42]. However, the current conventional pharmacological treatments for these diseases are not satisfactory. For instance, antibiotics or glucocorticoids used for peritonitis can quickly lead to drug resistance or systemic infection, and mesalazine or biological agents used for the treatment of IBD exhibit limited efficacy and fail to meet medical requirements [43,44]. Furthermore, prolonged use of these drugs often results in patient intolerance, accompanied by significant adverse reactions.

IRAK4 plays a crucial role in the intracellular signaling pathway, typically considered the downstream pathway of TLR or IL-1R, which regulates the transcription of inflammatory factors such as TNF-α and IL-6 and participates in the development of various inflammatory diseases [5]. In recent years, numerous small-molecule IRAK4 inhibitors have been designed, demonstrating their ability to effectively inhibit inflammatory signaling transduction and reduce gouty or ischemic inflammation in vitro and in vivo [5,24]. Pharmaceutical companies are vigorously developing potent and safe IRAK4 inhibitors for the treatment of various clinical conditions, including RA [22,45], systemic lupus erythematosus (SLE) [46,47], inflammatory dermatitis [45], the activated B-cell-like (ABC) subtype of diffuse large B-cell lymphoma (DLBCL) [45], and marginal zone lymphomas (MZL) [48]. Of particular note is the critical role of IRAK4 in regulating macrophages, making it a significant therapeutic target for various inflammatory diseases. However, as of now, no drug has received FDA approval, and there is a lack of clear clinical progress in the treatment of peritonitis and IBD. In light of this, we developed a novel IRAK4 inhibitor and investigated its potential in the treatment of peritonitis and inflammatory bowel disease.

In this study, we identified a novel IRAK4 inhibitor, DW18134. It exhibited significant kinase activity with an IC_50_ value of 11.2 nM, comparable to the IC_50_ value reported for the previously mentioned IRAK4 inhibitor PF-06650833 [23]. DW18134 showed significant anti-inflammatory activity in macrophages in vitro, inhibiting the phosphorylation of IRAK4 and its downstream molecule IKK and suppressing the secretion of cytokines in a dose-dependent manner. The results strongly supported the significant IRAK4 targeting and anti-inflammatory activity of DW18134. In addition, we also compared DW18134 with PF-06650833. Although the kinase activity of DW18134 was not better than PF-06650833, it was similar to PF-06650833 in the inhibition of the target and the secretion of cytokines at the cellular level. Our study is the first to assess the efficacy of DW18134 in two inflammatory disease animal models. As expected, DW18134 exhibited remarkable therapeutic effects in both LPS-induced peritonitis and DSS-induced IBD animal models. In the peritonitis mouse model characterized by a macrophage phenotype, DW18134 effectively reduced the behavioral score of the peritonitis mice and the secretion of inflammatory factors TNF-α, IL-6, and IL-1β in the serum, with therapeutic efficacy comparable to PF-06650833. In addition, DW18134 demonstrated significant therapeutic effects in acute injury models of the bowel, lung, and liver, concurrently reducing inflammatory gene expression. Notably, DW18134 effectively inhibited the phosphorylation of IRAK4 and its downstream molecules IKK and p65. Conversely, PF-06650833 did not exhibit a significant inhibitory effect on the activation of these proteins.

We were fully aware of the significance of our dose design in this research, and in formulating the dose regime for this study, we referred to the doses reported in Pfizer patent files [23,30], in which DW18134 has been verified to demonstrate outstanding efficacy. Significant therapeutic effects of DW18134 were also demonstrated in the DSS-induced IBD mouse model. DW18134 not only markedly reduced the DAI and alleviated body weight loss but also improved food and water intake in IBD mice, suggesting a significant therapeutic effect of DW18134 on IBD. IBD is closely associated with transmembrane inflammation and intestinal barrier imbalance. Surprisingly, we also found that DW181314 reversed the DSS-induced genetic alterations in tight junction proteins, such as ZO-1, E-cadherin, Occludin, and MUC2. The genes ZO-1, E-cadherin, and Occludin, associated with the tight junction, are closely related to intestinal barrier damage, and MUC2 is secreted by goblet cells. The expression of these genes was reversed by DW18134, indicating that DW18134 enables the restoration of intestinal barrier function and protects the loss of goblet cells, thereby reducing enteritis symptoms. We will continue to investigate how DW18134 regulates other tight junction proteins of the intestinal barrier in the future. Furthermore, our research results confirmed that DW18134 effectively reduced macrophage infiltration and inflammatory factor expression by targeting IRAK4 and inhibiting IKK activation. We designed a reasonable dose regimen to reflect the exact in vivo activity of DW18134 based on the reported dose in Pfizer patent documents [23], in which DW18134 has been proven to have excellent efficacy.

## 5. Conclusions

In conclusion, we identified a novel small-molecule IRAK4 inhibitor, DW18134, considered a promising candidate for the treatment of peritonitis and IBD. Our finding may provide a new strategy for the treatment of inflammatory diseases, with significant potential for clinical applications.

## Figures and Tables

**Table 1:** The IC_50_ values of compounds against IRAK4.

Compounds	DW18134	PF-06650833
Structure	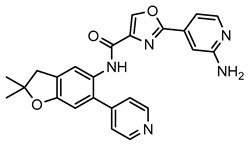	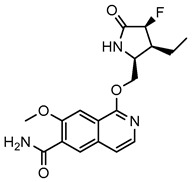
IC_50_ (nM)	11.2 ± 2.0	0.9 ± 0.1

## Data Availability

The data of this study are available from the corresponding authors upon request.

## Supplementary Materials

The following supporting information can be downloaded at https://www.mdpi.com/article/10.3390/molecules29081803/s1, Figure S1: Effects of PF-06650833 on the LPS-induced IRAK4 signaling transduction and secretion of cytokines in RAW264.7; Table S1: The behavior scoring standard of LPS-induced acute inflammation mouse model; Table S2: RT-qPCR primers used in this study; Table S3: Histological evaluations of H&E-stained.

## Abbreviations

BTK	Bruton’s tyrosine kinase
CD	Crohn’s disease
COPD	chronic obstructive pulmonary disease
DSS	dextran sulfate sodium salt
DAI	disease activity index
ELISA	enzyme linked immunosorbent assay
DLBCL	diffuse large B-cell lymphoma
IL-1β	interleukin 1 beta
IBD	inflammatory bowel disease
IL-6	interleukin-6
JAK	Janus kinase
LPS	lipopolysaccharide
IRAK4	interleukin receptor-associated kinase 4
IKK	inhibitor of Kappa B kinase
IL-1R	interleukin-1 receptor
Myd88	myeloid differentiation factor 88
MZL	marginal zone lymphomas
MUC2	mucoprotein
RT-qPCR	real-time quantitative PCR
RA	rheumatoid arthritis
SD	standard deviation
SBDD	Structure-based drug design
TNF-α	tumor necrosis factor alpha
TLR	toll-like receptor
UC	ulcerative colitis
ZO-1	zonula Occludin protein 1
