# Supplementary material for: A Novel IRAK4 Inhibitor DW18134 Ameliorates Peritonitis and Inflammatory Bowel Disease
Source: Molecules. 2024 Apr 16;29(8):1803. doi: 10.3390/molecules29081803 (PMC11052001; doi:10.3390/molecules29081803)
Supplement: Supplementary file 1 [file molecules-29-01803-s001.zip › molecules-2943449-supplementary.pdf]

Supplementary data

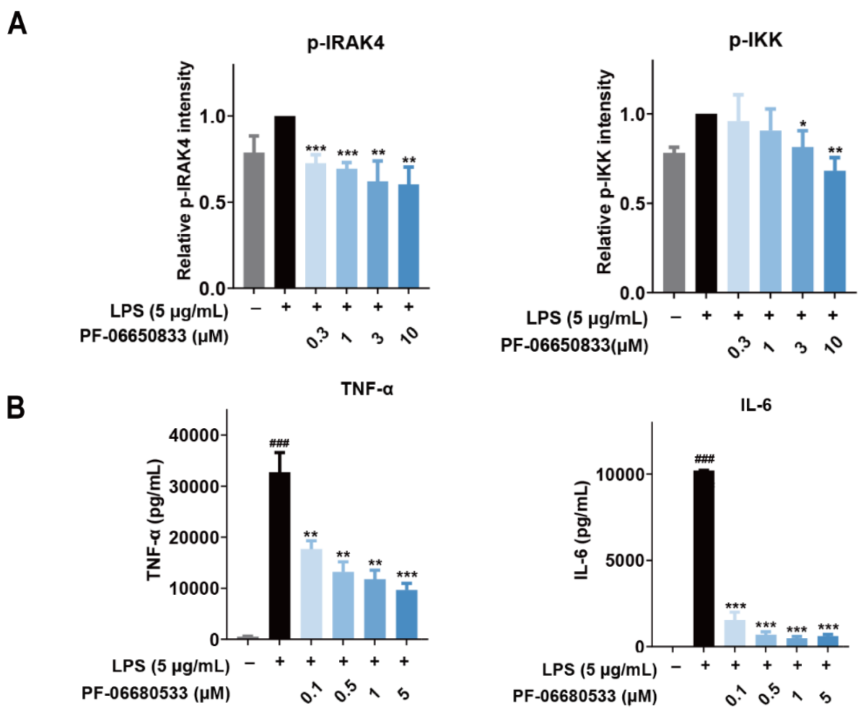

Figure S1: Effects of PF-06650833 on the LPS-induced IRAK4 signaling transduction and secretion of cytokines in RAW264.7. (A) Quantification of p-IRAK4 and p-IKK levels in RAW264.7 cells. (B) The secretion levels of TNF-a and IL-6 in the supernatants of RAW264.7 cells were measured by ELISA.

Table S1 The behavior scoring standard of LPS-induced acute inflammation mouse model

Score	0	1	2	3	4
Vertical hair	Asymptomatic	Mild	Medium	Serious	Extremely serious
Diarrhea	Asymptomatic	Mild	Medium	Serious	Extremely serious
Movement	Normal	Slightly Slow	Slow	Almost unchanged	No

Table S2 RT-qPCR primers used in this study

Gene		Sequence (5–3′)
TNF-α	Forward	GCTACGACGTGGGCTACAG
	Reverse	CCCTCACACTCAGATCATCTTCT
IL-6	Forward	AGTTGCCTTCTTGGGACTGA
	Reverse	CAGAATTGCCATTGCACAAC
IL-1β	Forward	GCAACTGTTCCTGAACTCAACT
	Reverse	ATCTTTTGGGGTCCGTCAACT
ZO-1	Forward	GCCGCTAAGAGCACAGCAA
	Reverse	TCCCCACTCTGAAAATGAGGA
Occludin	Forward	TTGAAAGTCCACCTCCTTA CAGA
	Reverse	CCGGATAAAAAGAGTACG CTGG
E-cadherin	Forward	CAGGTCTCCTCATGGCTTT GC
	Reverse	CTTCCGAAAAGAAGGCTG TCC
MUC2	Forward	AGGGCTCGGAACTCCAGA AA
	Reverse	CCAGGGAATCGGTAGACA TCG
Actin	Forward	GAGACCTTCAACACCCCAGC
	Reverse	ATGTCACGCACGATTTCCC

Table S3 Histological evaluations of H&E-stained

Score	symptom
0	no signs of inflammation
1	low leukocyte infiltration
2	moderate leukocyte infiltration
3	high leukocyte infiltration, moderate fibrosis, high vascular density, thickening of the colon wall, moderate goblet cell loss, and focal loss of crypts
4	transmural infiltrations, massive loss of goblet cells, extensive fibrosis, and diffuse loss of crypts
